# Efficacy of Elagolix and Relugolix for the Treatment of Pelvic Pain in Patients With Endometriosis: A Systematic Review

**DOI:** 10.7759/cureus.92149

**Published:** 2025-09-12

**Authors:** Mayuri Quishpe, Jesús Endara-Mina, Josselyn Caizapanta, Alisson Guzmán, Dayana Tenesaca, Diana Asto, Odalis Mena, Noelya Rengel, Jorge Avilés, Genesis Vaca, Henry Sarmiento-Vallejo

**Affiliations:** 1 Research, Escuela Superior Politécnica de Chimborazo, Riobamba, ECU; 2 Research, Eugenio Espejo Specialties Hospital, Quito, ECU; 3 Research, Universidad Regional Autónoma de Los Andes, Santo Domingo, ECU; 4 Research, Ministry of Public Health, Loja, ECU; 5 Research, Ministry of Public Health, Quito, ECU; 6 Obstetrics and Gynecology, University of the Americas, Quito, ECU

**Keywords:** dysmenorrhea, elagolix, endometriosis pain, ob-gyn, pelvic pain, relugolix

## Abstract

Endometriosis is a chronic gynecological condition commonly associated with pelvic pain, dysmenorrhea, dyspareunia, and infertility. Owing to the limitations and adverse effects of traditional hormonal therapies, this study aimed to evaluate the efficacy and safety of elagolix and relugolix for the management of endometriosis-related pain, in comparison with other therapeutic alternatives. A systematic search was conducted in PubMed/Medical Literature Analysis and Retrieval System Online (MEDLINE), Cochrane, SciELO, ScienceDirect, and Google Scholar, including randomized clinical trials (RCTs) published between 2014 and 2025. Methodological quality was assessed using Preferred Reporting Items for Systematic Reviews and Meta-Analyses (PRISMA) guidelines, together with the GRADE and Jadad tools. Nine clinical trials met the inclusion criteria. The findings suggest that both relugolix and elagolix, whether administered as monotherapy or in combination with estrogens and progestins, effectively reduce endometriosis-associated pain, improve the quality of life, and decrease the need for opioid use. Reported adverse events were generally mild, with minimal loss of bone mineral density (BMD). Importantly, the strongest and most consistent evidence supports the use of relugolix in combination with add-back therapy, which appears to provide sustained efficacy and a more favorable long-term safety profile than monotherapy. Thus, relugolix with hormonal add-back therapy emerges as a safe and effective long-term treatment option for endometriosis-related symptoms, offering significant clinical benefits while mitigating hypoestrogenic side effects.

## Introduction and background

Endometriosis is a chronic inflammatory gynecological condition characterized by the ectopic presence of functional endometrial tissue outside the uterine cavity, which responds to hormonal stimuli similarly to eutopic endometrium [1-3]. The most common clinical manifestation is chronic pelvic pain, considered the cardinal symptom, although it may also present with dysmenorrhea, dyspareunia, dyschezia, dysuria, and infertility [1]. This pathology affects approximately 6%-10% of women of reproductive age worldwide, with significantly higher rates in specific subgroups, such as those experiencing infertility (up to 50%) or chronic pelvic pain (up to 70%) [4,5]. According to the World Health Organization, around 190 million women globally live with endometriosis, making it one of the leading causes of noninfectious gynecological disability and having a substantial impact on the quality of life [6].

In Latin America, particularly in countries such as Ecuador, the true burden of the disease remains poorly characterized due to a scarcity of epidemiological studies, limited diagnostic capacity, and a lack of population registries. Hospital-based research in cities such as Quito, Guayaquil, and Cuenca has estimated that between 10% and 12% of women presenting with pelvic pain may suffer from endometriosis, with a reported diagnostic delay ranging from five to 10 years from the initial onset of symptoms [7,8]. This delay is exacerbated by sociocultural factors, including the stigma associated with severe menstrual pain, the normalization of symptoms, and barriers to accessing specialized gynecological care, disproportionately affecting women from rural areas, indigenous populations, and low-income groups [9-11].

Conventional therapeutic approaches are based on three main strategies: analgesic treatment, combined hormonal contraceptives, and gonadotropin-releasing hormone (GnRH) agonists, such as leuprolide, which induce a transient hypoestrogenic state to reduce lesion activity and alleviate pain [12,13]. However, the prolonged use of GnRH agonists is limited by significant adverse effects, such as severe climacteric symptoms, the loss of bone mineral density (BMD), and symptomatic recurrence after treatment cessation, while conventional analgesics provide incomplete relief in most cases [14,15]. In this context, GnRH antagonists represent a significant advancement over existing therapies. Unlike agonists, which initially stimulate a surge in gonadotropins before downregulating the receptor, antagonists act directly and immediately to suppress gonadotropin release without the initial flare effect.

Relugolix and elagolix, both oral GnRH receptor antagonists, have emerged as innovative therapeutic alternatives with potential for managing moderate to severe pain in patients with endometriosis. Their mechanisms of action directly and reversibly block the binding of GnRH to its pituitary receptors, thereby preventing the initial increase in gonadotropin characteristic of agonists and favoring a more favorable safety profile [16,17]. Given the need for effective and safe therapeutic options, this study aims to evaluate the efficacy of both relugolix and elagolix in reducing pain in patients with endometriosis, considering clinical benefits and the incidence of adverse effects. This will be achieved through a systematic review of recent randomized clinical trials (RCTs), providing updated and high-quality evidence to support clinical decision-making.

## Review

Methodology

The research question was structured using the participants, intervention, comparison, and outcome (PICO) framework, where the population consisted of adult women with a confirmed diagnosis of endometriosis; the intervention involved the administration of relugolix or elagolix, either as monotherapy or in combination with other therapies; the comparator was placebo or alternative medical treatment; and the primary outcome was the reduction in pain intensity. Secondary outcomes included improvement in the quality of life and the incidence of adverse effects.

This review was conducted following the guidelines of the Preferred Reporting Items for Systematic Reviews and Meta-Analyses (PRISMA) 2020 statement [18]. This study was registered in the International Prospective Register of Systematic Reviews (PROSPERO) under the identification number CRD420251118244, with registration completed on August 2, 2025. The registration was performed retrospectively, after the initiation of the systematic review process.

Search Strategy

A comprehensive and systematic literature search was conducted in the databases PubMed/Medical Literature Analysis and Retrieval System Online (MEDLINE), ScienceDirect, Cochrane Library, SciELO, and Google Scholar. The search strategy incorporated Medical Subject Headings (MeSH) terms, free-text terms, and synonyms to ensure a thorough retrieval of relevant studies. Boolean operators were used to combine the following concepts: efficacy, endometriosis, relugolix or elagolix, benefits, and adverse effects. The search string included both controlled vocabulary and open terms, for example, (efficacy OR effectiveness) AND (endometriosis) AND (relugolix OR elagolix OR GnRH antagonists) AND (benefits OR outcomes) AND (adverse effects OR side effects). The search was limited to publications in Spanish or English, published between January 2014 and June 2025. Additionally, a manual search was conducted by reviewing the reference lists of relevant articles. All retrieved references were manually reviewed to eliminate duplicates. The complete search strings are provided in the Appendices.

Inclusion and Exclusion Criteria

Randomized clinical trials (RCTs) with double masking that evaluated relugolix or elagolix for the management of pain associated with endometriosis, with a minimum follow-up of three months, were included. Studies that did not report pain outcomes, presented conflicts of interest that compromised the interpretation of results, or did not clearly specify the treatment regimen were excluded. Non-randomized trials, observational studies, reviews, editorials, and letters to the editor were also excluded.

Study Selection

The review of titles and abstracts was conducted independently by two reviewers (MQ and DT) to determine the eligibility of the studies. In cases of doubt, the full text was obtained for evaluation. Discrepancies were resolved by consensus or with the intervention of a third reviewer (HSV). After applying the selection criteria, a total of nine studies were included in the qualitative synthesis.

Data Extraction and Management

Data extraction was performed using a standardized form adapted from the model proposed by the Cochrane Consumers and Communication Review Group. The variables collected included author, the year of publication, journal, country and study setting, total sample size and by group, details of the intervention, the duration of follow-up, primary and secondary outcomes, adverse events, and ethical considerations (approval by an ethics committee and the inclusion of informed consent). Two reviewers conducted the extraction independently (JC and AG), resolving discrepancies by consensus or, if necessary, through a third reviewer (JEM).

Data Analysis and Synthesis

Due to the clinical and methodological heterogeneity of the included studies, particularly regarding treatment duration, pain measurement scales, and population characteristics, a meta-analysis was not performed. Instead, a qualitative narrative synthesis was conducted, grouping results by sample, follow-up time, intervention, and the type of outcome (pain reduction, quality of life improvement, and adverse effects).

Assessment of Methodological Quality

The quality of the included trials was assessed using the Jadad scale [19] and the GRADE approach [20] to evaluate the certainty of the evidence. Only studies with a score of ≥3 on the Jadad scale and at least a moderate level of certainty according to GRADE were included in the final synthesis. The risk of bias was analyzed based on the domains established by GRADE (selection bias, performance bias, detection bias, attrition bias, and reporting bias). Additionally, the Risk of Bias (RoB) II screening tool by Cochrane was used to assess risk of bias. To ensure transparency and reproducibility, a checklist based on the PRISMA 2020 guidelines was applied at all stages of the process. The details of the RoB II assessment are provided in the Appendices.

Results

**Figure 1:**
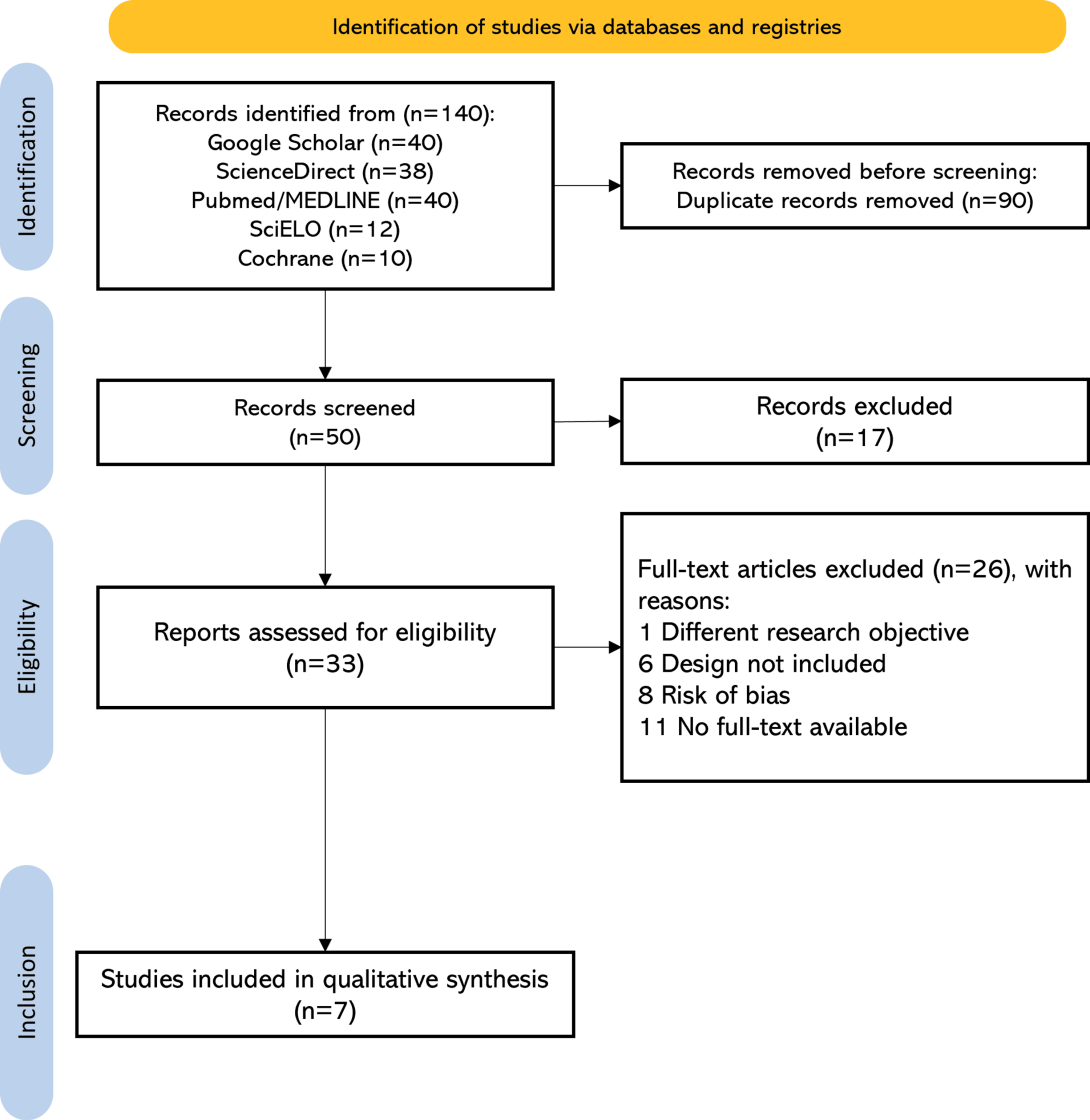
PRISMA flowchart for the identification and selection of included studies PRISMA, Preferred Reporting Items for Systematic Reviews and Meta-Analyses; MEDLINE, Medical Literature Analysis and Retrieval System Online

Through the application of specific search strategies and selection criteria detailed in Figure 1, a total of 140 articles were initially identified, with 90 duplicate records removed, resulting in 50 records for screening. Ten were excluded due to article type, and seven records were excluded for incomplete access. A total of 33 full-text articles were evaluated according to the inclusion criteria. Ultimately, nine randomized clinical trials were included in the qualitative synthesis, published in both Spanish and English (Figure 1).

Synthesis of Results

The studies included in this systematic review were published between 2014 and 2024, comprising a total of nine randomized clinical trials (RCTs), with a global sample of over 8,000 women diagnosed with endometriosis. The research was conducted in countries such as the United States, Japan, Canada, Brazil, the United Kingdom, Poland, Switzerland, Ukraine, Australia, and South Africa. The largest proportion of publications came from the United States and Japan, followed by collaborative trials in Europe and Latin America.

The variables frequently evaluated in each of the RCTs included dysmenorrhea, non-menstrual pelvic pain (NMPP), dyspareunia, opioid use, analgesic use, bone mineral density (BMD), adverse effects, and the quality of life through Endometriosis Health Profile-30 items (EHP-30) [21] and Uterine Fibroid Symptom and Quality of Life Questionnaire (UFS-QOL) [22] questionnaires.

Specifically, eight studies evaluated parameters related to pelvic pain (dysmenorrhea, non-menstrual pain, and dyspareunia) using validated scales such as the visual analog scale (VAS) [23] and numeric rating scale (NRS) [24]. Six trials included quality of life questionnaires, highlighting the EHP-30 and UFS-QOL, which showed clinically significant improvements in the domains of pain, emotional well-being, and control.

Seven of the nine studies employed combination therapy with relugolix, while two explored its comparison with GnRH analogs such as leuprolide. Additionally, five studies analyzed the use of both opioids and analgesics, demonstrating a sustained reduction in their consumption during and after treatment.

On the other hand, four trials assessed the effect of treatment on bone mineral density, with only mild loss observed, especially when relugolix was used in combination with estrogens and progestogens. Finally, seven studies reported adverse events, primarily hot flashes, nausea, and headaches, with no major clinical compromise or frequent need for treatment discontinuation.

The methodological quality criteria (Jadad scale) were adequate in all studies (≥3 points), and the certainty of evidence, according to the GRADE system, was classified as high in seven studies and moderate in two.

Table 1 presents the main characteristics of the included primary studies, specifying relevant information such as author, the year of publication, the country of conduct, trial title, methodological design, sample size, the duration of follow-up, the type of intervention, and main findings obtained [25-33].

**Table 1:** Randomized clinical trials used in the systematic review GnRH, gonadotropin-releasing hormone; QD, once daily; BID, twice daily; DMPA-SC, subcutaneous depot medroxyprogesterone acetate; BMD, bone mineral density; NS, not significant; RR, relative risk; VAS, visual analog scale; QoL, quality of life; EHP-30, Endometriosis Health Profile-30 items; CT, combination therapy

Number	Author, Year, and Country	Trial Title	Design	Sample and Follow-Up	Intervention	Variables	Results	Analysis	GRADE/Jadad
1	Carr et al., 2014 (United States) [25]	Elagolix, an Oral GnRH Antagonist, Versus Subcutaneous Depot Medroxyprogesterone Acetate for the Treatment of Endometriosis: Effects on Bone Mineral Density	Phase 2, randomized, double-blind, placebo-controlled trial	252 patients (elagolix, 126; placebo, 126). Follow-up: 24 weeks	Elagolix 150 mg QD or 75 mg BID versus DMPA-SC	Bone mineral density (lumbar spine and hip); non-menstrual pelvic pain	Minimal, nonsignificant BMD loss across groups. Sustained pelvic pain improvement; 86% clinical response with elagolix 150 mg versus 76.5% with DMPA-SC (NS)	Comparable efficacy with minimal skeletal impact	High; 5/5
2	Taylor et al., 2017 (multinational) [26]	Treatment of Endometriosis-Associated Pain with Elagolix, an Oral GnRH Antagonist	Phase 3, randomized, double-blind, placebo-controlled, multicenter trial	1689 patients (elagolix, 872; placebo, 817). Follow-up: 6-12 months	Elagolix 150 mg QD or 200 mg BID versus placebo	Dysmenorrhea, pelvic pain, dyspareunia, and opioid use	Significant reduction in dysmenorrhea (RR, 2.4-3.9; p<0.001) and pelvic pain (RR, 1.3-1.5; p<0.001). Dyspareunia improved with 200 mg BID. Opioid use decreased modestly	High-dose elagolix demonstrated superior clinical efficacy	High; 5/5
3	Surrey et al., 2018 (multinational) [27]	Long-Term Outcomes of Elagolix in Women With Endometriosis: Results From Two Extension Studies	Phase 3, randomized, double-blind, placebo-controlled trial (extension EM-I/II)	569 patients (elagolix, 282; placebo, 287). Follow-up: 12 months	Elagolix 150 mg QD or 200 mg BID versus placebo	Dysmenorrhea, pelvic pain, dyspareunia, and analgesic use	Sustained reduction in dysmenorrhea (>75% responders at 12 months). Consistent effect on pelvic pain. Dyspareunia and analgesic use also improved	Sustained efficacy with reduced need for concomitant medication	Moderate; 4/5
4	Osuga et al., 2021 (Japan) [28]	Relugolix, an oral gonadotropin-releasing hormone receptor antagonist, reduces endometriosis-associated pain in a dose–response manner: a randomized, double-blind, placebo-controlled study	Phase 3, randomized, double-blind, placebo-controlled trial	487 patients (relugolix, 243; placebo, 244). Follow-up: 12+4 weeks	Relugolix 20 or 40 mg QD versus placebo	Pelvic pain (VAS), QoL (EHP-30), BMD, and analgesic use	Dose-dependent pain reduction: -6.2 mm (20 mg) and -4.3 mm (40 mg) versus placebo (p<0.05). QoL improved. Minimal BMD loss	Comparable to leuprorelin, with a faster onset and no flare-up	High; 5/5
5	Harada et al., 2022 (Japan) [29]	Relugolix, an oral gonadotropin-releasing hormone receptor antagonist, reduces endometriosis-associated pain compared with leuprorelin in Japanese women: a phase 3, randomized, double-blind, noninferiority study	Phase 3, randomized, double-blind, non-inferiority trial	335 patients (relugolix, 167; leuprorelin, 168). Follow-up: 24+4 weeks	Relugolix 40 mg QD versus leuprorelin injections	Pelvic pain, dysmenorrhea, analgesic use, QoL, and menstruation recovery	Relugolix was non-inferior for pain relief. Faster menstruation recovery (38 versus 68 days). QoL improved. Mild BMD reduction, comparable across groups	Effective alternative, especially for women with reproductive goals	High; 5/5
6	Giudice et al., 2022 (multinational) [30]	Once daily oral relugolix combination therapy versus placebo in patients with endometriosis-associated pain: two replicate phase 3, randomised, double-blind, studies (SPIRIT 1 and 2)	Phase 3, randomized, double-blind, placebo-controlled trial	1261 patients (relugolix CT, 424; placebo, 425). Follow-up: 24 weeks	Relugolix 40 mg+estradiol 1 mg+norethisterone acetate 0.5 mg QD versus placebo	Dysmenorrhea, pelvic pain, opioid use, and adverse events	Significant reductions in dysmenorrhea (≥45%) and pelvic pain (18%-23%) versus placebo (p<0.0001). Opioid use substantially reduced. Adverse events similar to placebo; BMD loss of <1%	Robust efficacy with favorable long-term safety	High; 5/5
7	As-Sanie et al., 2024 (United States and multinational) [31]	Impact of relugolix combination therapy on functioning and quality of life in women with endometriosis-associated pain	Phase 3, randomized, double-blind, placebo-controlled, multicenter trial	802 patients (relugolix CT, 401; Placebo, 401). Follow-up: 104 weeks	Relugolix CT (40 mg relugolix, 1 mg estradiol, and 0.5 mg norethisterone acetate) versus placebo	QoL (EHP-30), dysmenorrhea, pelvic pain, and BMD	Significant and sustained QoL improvement (EHP-30 pain, -32.8 to -41.3; p<0.0001); ≥20-point pain reduction in 88.6% at 104 weeks. BMD loss of <1%	Long-term efficacy with durable safety	High; 5/5

The detailed extensions of the SPIRIT 1 and 2 trials, as well as the ELARIS EM-I and EM-II studies, are provided in the Appendices to allow the comprehensive consultation of their long-term efficacy and safety data.

Discussion

This systematic review, which integrates evidence from nine randomized clinical trials with a large sample of women diagnosed with endometriosis, provides a comprehensive assessment of the clinical efficacy, safety, impact on quality of life, and reduction in analgesic use of treatment with relugolix and elagolix, both as monotherapy and in combination with hormonal therapy.

The findings confirm that relugolix, whether administered alone or in conjunction with estrogens and progestogens, represents an effective and well-tolerated therapeutic alternative for the treatment of pain associated with endometriosis. Across the included clinical trials [25-31], between 70% and 88.6% of patients achieved clinically significant improvement in pelvic pain control, as measured using validated tools such as the EHP-30 [22] and NRS [24], over follow-up periods ranging from 24 to 104 weeks. In SPIRIT 1 and 2, a 75% reduction in dysmenorrhea and a 59%-66% reduction in non-menstrual pelvic pain (NMPP) were observed, with an absolute difference of 47.6% (95% CI: 39.3-56.0; p<0.0001) compared to placebo [30]. Opioid use decreased by up to 15.9% in the treatment group, without clinically relevant changes in bone mineral density (BMD) or serious adverse events. Dyspareunia also improved in several trials, with Taylor et al. (2017) [26] and Surrey et al. (2018) [27] reporting up to a 60% reduction, compared to 36.5% with placebo (p<0.001), and relative risks ranging from 1.4 to 1.6. Bone safety outcomes were favorable, with combination therapy maintaining BMD loss under 1% even at 104 weeks, according to As-Sanie et al. (2024) [31] and Osuga et al. (2021) [28]. Mild but significant reductions in lumbar spine BMD (-2.1% versus -0.1% with placebo; p<0.005) were observed but without the increased incidence of hot flashes or flare-up phenomena commonly associated with GnRH agonists [25-31].

These findings are consistent with previous meta-analyses. For example, Sobral et al. (2025) demonstrated an 84% reduction in dysmenorrhea and a 68.9% reduction in non-menstrual pain after two years of treatment, with sustained long-term benefits [32]. These results support the hypothesis that the analgesic effect may be mediated by the stable modulation of the hypothalamic-pituitary-ovarian axis, leading to suppressed estradiol levels without inducing severe hypoestrogenism [33]. Similarly, Xin et al. (2023) found that GnRH antagonists significantly reduced pelvic pain (relative risk {RR}, 0.48; 95% CI, 0.35-0.65), with less bone loss compared to GnRH agonists such as leuprolide [34]. On the other hand, the Cochrane meta-analysis by Veth et al. (2023), which included 15 randomized trials and over 1,800 women, concluded that while GnRH agonists (e.g., leuprolide and nafarelin) are effective for pain relief, they are limited by adverse effects such as hypoestrogenism-induced BMD loss, severe hot flashes, mood disturbances, and vaginal dryness [35]. These effects restrict their use to six months or require co-treatment with hormone replacement therapy [35,36].

Overall, the studies included in this review, especially SPIRIT 1 and 2, suggest that relugolix combination therapy can be administered for up to 24 months without significant bone loss (<1%) while maintaining efficacy in dysmenorrhea, NMPP, and reduction in opioid use [30]. This could represent a potential shift in the therapeutic paradigm, enabling longer-term treatment for women with endometriosis who require sustained pain control, particularly those of reproductive age.

Regarding non-menstrual pelvic pain, the SPIRIT 2 trial, with 623 participants, reported improvement in 66% of treated patients compared to 43% with placebo, with an absolute difference of 23.4% (95% CI, 14.0-32.8; p<0.0001) [30]. Studies by Harada et al. (2022) [29] and As-Sanie et al. (2024) [31] confirm that, although NMPP has a more complex pathophysiology, relugolix maintains a sustained effect, particularly in combination therapy, with VAS differences ranging from -4.3 to -6.2 mm compared to placebo.

In terms of dyspareunia, while Vercellini et al. (2016) [37] noted that this symptom tends to be less responsive to hormonal treatment, Taylor et al. (2017) [26] and Surrey et al. (2018) [27] demonstrated notable improvements with relugolix, suggesting that its therapeutic effects may extend beyond general pelvic pain to symptoms that directly impact sexual quality of life.

Regarding bone mineral density, a common concern in estrogen-suppressive therapies, the analyzed studies demonstrate that the combination of relugolix with hormonal therapy offers adequate protection, with losses of <1% even at 104 weeks [38]. This contrasts with the accelerated bone loss typically seen with GnRH agonists and supports the long-term safety profile of relugolix-based regimens [35,36].

Limitations

Some multinational trials included heterogeneous populations in terms of age, the stage of endometriosis, and prior treatments, which hinder the extrapolation of results to specific groups such as adolescents, perimenopausal women, or individuals with infertility [30]. In addition, some studies applied strict exclusion criteria, omitting patients with gynecological comorbidities such as fibroids and adenomyosis, which are common in clinical practice, thereby limiting the applicability of findings to more complex scenarios. Differences in follow-up duration (ranging from 12 to 104 weeks) may also affect the comparability of outcomes such as bone mineral density, chronic dysmenorrhea, and adverse events. Standardizing study duration and incorporating post-treatment follow-up would be desirable to better assess the persistence of effects.

Furthermore, if PROSPERO registration had been performed retrospectively, this could raise concerns about selective reporting bias. Although such bias cannot be entirely excluded, the risk was mitigated by maintaining consistency between predefined and reported outcomes and ensuring that all methodological deviations were transparently documented. Finally, articles published in languages other than English or Spanish were excluded due to operational limitations in technical translation, which may have introduced a degree of language bias.

## Conclusions

This systematic review highlights that relugolix and elagolix, used either as monotherapy or in combination with add-back therapy, are effective and well-tolerated pharmacological options for the management of endometriosis-associated pain. Evidence from randomized clinical trials consistently demonstrates meaningful improvements in core symptoms, contributing to enhanced daily functioning and quality of life for affected women.

Furthermore, these treatments offer advantages beyond symptom control, including a reduced need for concomitant analgesics, which underscores their relevance in long-term pain management strategies. While current findings support their integration into clinical practice, further research is necessary to confirm their long-term safety and effectiveness, ensuring their sustained role in the therapeutic landscape of endometriosis.

## Appendices

Table 2 shows the extent of the SPIRIT and ELARIS EM studies.

**Table 2:** Extension of SPIRIT and ELARIS EM studies RCT: relugolix combination therapy

Number	Author, Year, and Country	Trial Title	Design	Sample and Follow-up	Intervention	Outcomes	Results	Analysis	GRADE/Jadad
1	Giudice et al., 2022 (multinational) (SPIRIT 1) [30]	Relugolix combination therapy with estradiol and norethisterone acetate in women with endometriosis-associated pain (SPIRIT 1)	Phase 3, multicenter, randomized, double-blind, placebo-controlled trial	638 women (RCT, 321; placebo, 317). Follow-up: 24 weeks	Relugolix 40 mg+estradiol 1 mg+norethisterone acetate 0.5 mg once daily versus placebo	Menstrual pain, non-menstrual pelvic pain, the quality of life, and analgesic use	Significant reduction in both menstrual and non-menstrual pain compared to placebo; decreased reliance on analgesics; clinically meaningful improvements in the quality of life	Demonstrated high efficacy with a consistent safety profile	High; 5/5
2	Giudice et al., 2022 (multinational) (SPIRIT 2) [30]	Relugolix combination therapy with estradiol and norethisterone acetate in women with endometriosis-associated pain (SPIRIT 2)	Phase 3, multicenter, randomized, double-blind, placebo-controlled trial	623 women (RCT, 312; placebo, 311). Follow-up: 24 weeks	Relugolix 40 mg+estradiol 1 mg+norethisterone acetate 0.5 mg once daily versus placebo	Menstrual pain, non-menstrual pelvic pain, the quality of life, and analgesic use	Findings consistent with SPIRIT 1: significant improvement in pain reduction, reduced need for concomitant analgesics, and favorable safety profile	High efficacy with reproducible results across populations	High; 5/5
3	Taylor et al., 2017 (multinational) (ELARIS EM-I) [26]	Elagolix for endometriosis-associated pain (ELARIS EM-I)	Phase 3, multicenter, randomized, double-blind, placebo-controlled trial	872 women. Follow-up: six months	Elagolix 150 mg once daily or 200 mg twice daily versus placebo	Menstrual pain, non-menstrual pelvic pain, analgesic use, and the quality of life	Both elagolix regimens significantly reduced menstrual and non-menstrual pain versus placebo; decreased analgesic use; dose-dependent hypoestrogenic adverse effects observed	Robust efficacy with a manageable safety profile	High; 5/5
4	Taylor et al., 2017 (multinational) (ELARIS EM-II) [26]	Elagolix for endometriosis-associated pain (ELARIS EM-II)	Phase 3, multicenter, randomized, double-blind, placebo-controlled trial	817 women. Follow-up: six months	Elagolix 150 mg once daily or 200 mg twice daily versus placebo	Menstrual pain, non-menstrual pelvic pain, analgesic use, and the quality of life	Consistent with EM-I: significant reductions in both pain domains, lower analgesic use, and a safety profile marked by mild-to-moderate hypoestrogenic effects and bone mineral density loss	Confirmed efficacy and reproducibility across study populations	High; 5/5

Table 3 shows the search equations.

**Table 3:** Search equations MEDLINE: Medical Literature Analysis and Retrieval System Online

Databases	Search Equations
PubMed/MEDLINE	("Endometriosis"[Mesh] OR endometriosis) AND ("Pelvic Pain"[Mesh] OR "Dysmenorrhea"[Mesh] OR "Dyspareunia"[Mesh] OR pelvic pain OR pelvic pains OR pelvic-pain OR dysmenorrhea OR dysmenorrhoeas OR dyspareunia OR "nonmenstrual pelvic pain" OR "non-menstrual pelvic pain") AND ("Elagolix" OR "Relugolix" OR "Gonadotropin-Releasing Hormone Antagonists" OR "GnRH antagonist" OR "oral GnRH antagonist") AND ("Treatment Outcome"[Mesh] OR "Patient Reported Outcome Measures"[Mesh] OR "Quality of Life"[Mesh] OR "Visual Analog Scale"[Mesh] OR efficacy OR effectiveness OR benefit OR "pain reduction" OR "pain relief" OR "quality of life" OR "Drug-Related Side Effects and Adverse Reactions"[Mesh] OR safety OR tolerability OR "adverse effect" OR "adverse event") AND ("2014/01/01"[Date - Publication]: "2025/06/30"[Date - Publication]) AND (english[lang] OR spanish[lang]) AND humans[MeSH Terms]
Cochrane	(MeSH descriptor: [Endometriosis] explode all trees OR endometriosis:ti,ab,kw) AND (MeSH descriptor: [Pelvic Pain] explode all trees OR MeSH descriptor: [Dysmenorrhea] explode all trees OR MeSH descriptor: [Dyspareunia] explode all trees OR pelvic pain OR dysmenorrhea OR dyspareunia OR "nonmenstrual pelvic pain" OR "non-menstrual pelvic pain") AND (MeSH descriptor: [Gonadotropin-Releasing Hormone Antagonists] explode all trees OR elagolix OR relugolix OR "GnRH antagonist" OR "gonadotropin-releasing hormone antagonist" OR "oral GnRH antagonist") AND (MeSH descriptor: [Treatment Outcome] explode all trees OR MeSH descriptor: [Patient Reported Outcome Measures] explode all trees OR MeSH descriptor: [Quality of Life] explode all trees OR MeSH descriptor: [Drug-Related Side Effects and Adverse Reactions] explode all trees OR efficacy OR effectiveness OR benefit OR "pain reduction" OR "pain relief" OR "quality of life" OR safety OR tolerability OR "adverse effect" OR "adverse event")
SciELO	(endometriosis) AND ("dolor pélvico" OR dismenorrea OR dispareunia OR "dolor pélvico no menstrual") AND (elagolix OR relugolix OR "antagonista de GnRH" OR "antagonistas de GnRH" OR "antagonista oral de GnRH") AND (eficacia OR efectividad OR beneficios OR "resultado del tratamiento" OR "calidad de vida" OR seguridad OR "eventos adversos" OR tolerabilidad)
ScienceDirect	TITLE-ABSTR-KEY(endometriosis AND ("pelvic pain" OR dysmenorrhea OR dyspareunia OR "nonmenstrual pelvic pain" OR "non-menstrual pelvic pain") AND (elagolix OR relugolix OR "GnRH antagonist*" OR "gonadotropin-releasing hormone antagonist" OR "oral GnRH antagonist") AND (efficacy OR effectiveness OR benefit OR "treatment outcome" OR "quality of life" OR "pain reduction" OR "pain relief" OR safety OR tolerability OR "adverse effect" OR "adverse event"))
Google Scholar	("endometriosis") AND ("pelvic pain" OR dysmenorrhea OR dyspareunia OR "nonmenstrual pelvic pain" OR "non-menstrual pelvic pain") AND (elagolix OR relugolix OR "GnRH antagonist" OR "gonadotropin-releasing hormone antagonist" OR "oral GnRH antagonist") AND (efficacy OR effectiveness OR "treatment outcome" OR "quality of life" OR "pain reduction" OR safety OR tolerability OR "adverse effects" OR "adverse events")

Table 4 shows the analysis of the risk of bias in the randomized studies included with RoB II.

**Table 4:** Analysis of risk of bias in the randomized studies included with RoB II RoB: Risk of Bias

Study	Bias From the Randomization Process	Bias Due to Intended Interventions	Bias Due to Missing Outcome Data	Bias in the Measurement of the Outcomes	Bias in the Selection of the Reported Result	Overall Risk of Bias
Carr et al., 2014 (United States) [25]	Low risk	Low risk	Some concerns	Low risk	Low risk	Some concerns
Taylor et al., 2017 (multinational) [26]	Low risk	Low risk	Low risk	Low risk	Low risk	Low risk
Surrey et al., 2018 (multinational) [27]	Low risk	Some concerns	Some concerns	Low risk	Low risk	Some concerns
Osuga et al., 2021 (Japan) [28]	Low risk	Low risk	Low risk	Low risk	Low risk	Low risk
Harada et al., 2022 (Japan) [29]	Low risk	Low risk	Low risk	Low risk	Low risk	Low risk
Giudice et al., 2022 (multinational) [30]	Low risk	Low risk	Low risk	Low risk	Low risk	Low risk
As-Sanie et al., 2024 (United States and multinational) [31]	Low risk	Low risk	Low risk	Low risk	Low risk	Low risk
